# A Heart Rate Based Algorithm to Estimate Core Temperature Responses in Elite Athletes Exercising in the Heat

**DOI:** 10.3389/fspor.2022.882254

**Published:** 2022-06-22

**Authors:** Johannus Q. de Korte, Bertil J. Veenstra, Mark van Rijswick, Eline J. K. Derksen, Maria T. E. Hopman, Coen C. W. G. Bongers, Thijs M. H. Eijsvogels

**Affiliations:** ^1^Department of Physiology, Radboud University Medical Center, Radboud Institute for Health Sciences, Nijmegen, Netherlands; ^2^Institute of Training Medicine & Training Physiology, TGTF, Royal Netherlands Army, Utrecht, Netherlands

**Keywords:** validity, prediction, real-time monitoring, physiological thermal strain, sports

## Abstract

**Purpose:**

Non-invasive non-obtrusive continuous and real-time monitoring of core temperature (T_c_) may enhance pacing strategies, the efficacy of heat mitigation measures, and early identification of athletes at risk for heat-related disorders. The Estimated Core Temperature (ECTemp™) algorithm uses sequential heart rate (HR) values to predict T_c_. We examined the validity of ECTemp™ among elite athletes exercising in the heat.

**Methods:**

101 elite athletes performed an exercise test in simulated hot and humid environmental conditions (ambient temperature: 31.6 ± 1.0°C, relative humidity: 74 ± 5%). T_c_ was continuously measured using a validated ingestible telemetric temperature capsule system. In addition, HR was continuously measured and used to compute the estimated core temperature (T_c−est_) using the ECTemp™ algorithm.

**Results:**

Athletes exercised for 44 ± 10 min and *n* = 5,025 readouts of T_c_ (range: 35.8–40.4°C), HR (range: 45–207 bpm), and T_c−est_ (range: 36.7–39.9°C) were collected. T_c−est_ demonstrated a small yet significant bias of 0.15 ± 0.29°C (*p* < 0.001) compared to T_c_, with a limit of agreement of ±0.45°C and a root mean square error of 0.35 ± 0.18°C. Utilizing the ECTemp™ algorithm as a diagnostic test resulted in a fair to excellent sensitivity (73–96%) and specificity (72–93%) for T_c−est_ thresholds between 37.75 and 38.75°C, but a low to very-low sensitivity (50–0%) for T_c−est_ thresholds >39.0°C, due to a high prevalence of false-negative observations.

**Conclusion:**

ECTemp™ provides a valuable and representative indication of thermal strain in the low- to mid-range of T_c_ values observed during exercise in the heat. It may, therefore, be a useful non-invasive and non-obtrusive tool to inform athletes and coaches about the estimated core temperature during controlled hyperthermia heat acclimation protocols. However, the ECTemp™ algorithm, in its current form, should not solely be used to identify athletes at risk for heat-related disorders due to low sensitivity and high false-negative rate in the upper end of the T_c_ spectrum.

## Introduction

Climate change is projected to increase global ambient temperatures and increase the frequency, intensity, and duration of heat waves (Haines et al., 2006; Romanello et al., 2021). The higher means and extremes of ambient temperatures will particularly challenge (elite) athletes, as prolonged exercise, especially under heat stress, can produce profound elevations in core temperature (T_c_) (Racinais et al., 2019), potentially leading to attenuated exercise performance (de Korte et al., 2021a) and an increased risk for heat-related illnesses (American College of Sports Medicine et al., 2007; Epstein and Roberts, 2011; Casa et al., 2015; Periard et al., 2021; Bouchama et al., 2022). Exertional heat illness can vary from mild complaints such as exercise-associated muscle cramps, to more serious concerns such as heat syncope and heat exhaustion, and can also become life threatening during a heat stroke. Continuous and real-time monitoring of T_c_ allows early identification of excessive T_c_ perturbations and adequate health risk assessment for heat-related disorders, which opens new avenues to adjust pacing strategies and improve mitigation measures to attenuate T_c_ elevations (Casa et al., 2015; Racinais et al., 2015). Available methodologies to assess T_c_ in a sports setting are, however, limited due to impracticality (i.e., rectal and esophageal temperature) (Moran and Mendel, 2001), inaccuracy (i.e., tympanic and axillary temperature) (Casa et al., 2007), or relatively high costs (i.e., temperature capsule). While the field of wearable technology for monitoring human vital signs has developed rapidly in recent years (Khan et al., 2016), real-time monitoring of T_c_ during exercise remains challenging and the need for simple and non-invasive non-obtrusive measures to monitor T_c_ is warranted.

In the last decades, several attempts have been made to non-invasively predict T_c_ using single or multiple physiological parameters (i.e., heart rate, skin temperature, heat flux) (Yokota et al., 2008; Buller et al., 2013; Niedermann et al., 2014; Richmond et al., 2015; Eggenberger et al., 2018; Welles et al., 2018; Moyen et al., 2021; Verdel et al., 2021). The Estimated Core Temperature algorithm (ECTemp™) was developed to estimate T_c_ based on sequential heart rate (HR) observations alone using a Kalman filter (Kalman, 1960) and a sigmoid curve (Looney et al., 2018). ECTemp™ has been shown to provide an accurate indication of thermal strain in military personnel during moderate-intensity activities (i.e., road march) and endurance exercise (up to 24 h) in laboratory- and field settings (Buller et al., 2013, 2018, 2020). Nevertheless, the validity of the ECTemp™ algorithm in (elite) athletes has not been evaluated yet. Since factors that challenge thermal homeostasis (i.e., clothing, exercise intensity, and duration) can be very different between military personnel and elite athletes (Ashworth et al., 2020), it remains unclear whether the ECTemp™ algorithm is a reliable tool to estimate T_c_ in elite athletes during exercise in the heat.

We aimed to determine the validity of the ECTemp™ algorithm to predict T_c_ of elite athletes performing exercise in hot and humid environmental conditions. We also explored the impact of sex and sport discipline on the validity of the ECTemp™ algorithm. Outcomes of this study can inform athletes and coaches whether ECTemp™ could be a useful technology to obtain real-time estimations of T_c_ during training and competition.

## Materials and Methods

### Participants

Dutch elite athletes ≥16 years old and practicing an outdoor sport discipline on an international level were eligible to participate in our study. Exclusion criteria were based on the use of the ingestible temperature capsule: (I) a bodyweight <36.5 kg, (II) an implanted electro-medical device, (III) a history of obstructive/inflammatory bowel disease or surgery, or (IV) a scheduled MRI scan within 5 days of the experiment. Participant characteristics of the analytical cohort (*n* = 101) as well as for groups based on sex and sport discipline are presented in Table 1. Participants were active in 13 different types of sport, including *n* = 27 endurance trained athletes (mountain biking *n* = 5, open water swimming *n* = 2, road cycling *n* = 7, triathlon *n* = 13), *n* = 28 mixed trained athletes (3 × 3 basketball *n* = 5, beach volleyball *n* = 8, field hockey *n* = 14, soccer *n* = 1), *n* = 11 power trained athletes (BMX *n* = 11), and *n* = 35 skill trained athletes (baseball *n* = 10, sailing *n* = 3, skateboarding *n* = 2, softball *n* = 20). None of the participating athletes conducted a dedicated heat acclimatization program prior to participation, and only four athletes reported having some heat exposure but were not acclimatized. The study was in accordance with the Declaration of Helsinki and was approved by the Medical Ethical Committee of the Radboud university medical center (#2018-4640). All participants gave their written informed consent prior to the testing procedures.

**Table 1:** Participant characteristics for the whole analytical cohort as well as for groups based on sex and sport disciplines.

	**Sex**		**Sport discipline**				**Analytical cohort**
	**Males (*n* = 49)**	**Females (*n* = 52)**	**Endurance trained athletes (*N* = 27)**	**Mixed trained athletes (*N* = 28)**	**Power trained athletes (*N* = 11)**	**Skill trained Athletes (*N* = 35)**	**All athletes (*N* = 101)**
Age (years)	26 ± 5	26 ± 5	25 ± 6	27 ± 4	23 ± 3	26 ± 5	26 ± 5
Sex (*n*, (% Male)			12 (44%)	20 (71%)	7 (64%)	10 (29%)	49 (49%)
Height (cm)	187 ± 9	172 ± 7	176 ± 10	186 ± 12	177 ± 7	177 ± 10	179 ± 11
Weight (kg)	83.2 ± 12.4	68.7 ± 10.2	65.5 ± 9.4	80.3 ± 11.5	80.1 ± 8.9	78.7 ± 14.7	75.8 ± 13.4
BMI (kg/m^2^)	23.8 ± 2.8	23.2 ± 2.7	20.9 ± 1.4	23.1 ± 1.4	25.6 ± 1.1	25 ± 3	23.4 ± 2.7
BSA (m^2^)	2.08 ± 0.19	1.81 ± 0.16	1.81 ± 0.18	2.05 ± 0.22	1.97 ± 0.15	1.95 ± 0.22	1.94 ± 0.22

*BMI, body mass index; BSA, body surface area. Data is presented as mean ± SD and range or n (%)*.

### Design

This study is part of the Thermo Tokyo research project, which rationale and design have been described in detail previously (de Korte et al., 2021b). In short, participants were invited to complete a personalized incremental exercise test on a cycling ergometer (Lode ergometer, Lode B.V., Groningen, Netherlands, or Tacx Neo Smart T2800, Tacx B.V., Wassenaar, Netherlands) in simulated hot and humid environmental conditions (ambient temperature 31.6 ± 1.0°C, relative humidity 74 ± 5%, Wet Bulb Globe Temperature 28.6 ± 0.8°C, ambient vapor pressure 3.45 kPa, absolute humidity 0.0245 kg/m^3^). The specific heat stress environmental conditions were chosen to simulate the heat stress expected for the Tokyo 2020 Olympic Games (Gerrett et al., 2019), which can be categorized as high according to previously described classifications based on the Wet Bulb Globe Temperature and relative humidity (Gonzalez, 1995; American College of Sports Medicine et al., 2007). Participants were instructed to refrain from strenuous exercise (24 h) and consumption of alcohol or caffeine (12 h) prior to the exercise test. Furthermore, all participants were instructed to consume their last meal ≥3 h preceding the experiment and consume 500 ml of water ~2 h before arriving at the laboratory. The exercise tests consisted of a 20 min warm-up at ±70% of the maximal HR, which was obtained from training data or a previously performed maximal exercise test. The warm-up phase was followed by an incremental phase during which the workload, measured in W, was increased by 5% every 3 min until volitional exhaustion. T_c_, HR, and power output were measured continuously throughout the protocol.

### Measurements

#### Anthropometrics

Body weight was measured to the nearest 100 g using an electronic weighing scale (Seca robusta 813 scale, Hamburg, Germany). Body height was measured to the nearest cm using a stadiometer (Road Rod Portable Stadiometer, Hopkins medical products, Caledonia, USA).

#### Exercise Performance

Peak power output was determined at the end of the exercise protocol and was expressed as an absolute (W) and normalized (W/kg) value.

#### Heart Rate

A 2-channel HR chest strap (Polar V800, Polar Electro Oy, Kempele, Finland) was used to measure HR at 1 s intervals throughout the exercise protocol.

#### Core Temperature

T_c_ served as the reference measurement and was continuously measured in Celsius at predefined 10 s intervals using a validated ingestible telemetric temperature capsule system (myTemp, Nijmegen, Netherlands) (Bongers et al., 2018a,b). Participants ingested the telemetric temperature capsule ~3 h prior to participation and were not allowed to drink during the exercise protocol to avoid any interaction with fluid intake (Wilkinson et al., 2008).

### Data Processing

After data extraction, individual data records were cleaned by manually removing erroneous outlier values for T_c_. Thereafter, min averages of T_c_ and HR were calculated using a customized MATLAB and Statistics Toolbox (2012b, The MathWorks, Inc., Natick, USA) software package. Missing T_c_ data were linearly interpolated using a customized MATLAB script if gaps were <5 min. Gaps >5 min were visually inspected by two researchers and only interpolated if the interpolated data fitted the T_c_ curve. In case of discrepancy between the two researchers, the evaluation of a third researcher was decisive. All case-by-case visual data inspections were performed by the same three dedicated and experienced researchers (JK, CB, TE). A total of 106 elite athletes completed the exercise protocol. T_c_ measurements were missing in 3 participants due to a loss of sensor signal and data records from another 2 participants were excluded based on significant abnormalities in the T_c_ curve following a case-by-case review. The analytical cohort consisted of 101 athletes.

#### Estimated Core Temperature (T_c-Est_)

The sequential min averages of HR were used to compute the estimated core temperature (T_c−est_) according to the updated sigmoid curve ECTemp™ algorithm (Looney et al., 2018; Buller et al., 2020). The original ECTemp™ algorithm used an extended Kalman Filter (Buller et al., 2013) and was updated with a sigmoid curve to better represent the relationship between HR and T_c_ (Looney et al., 2018; Buller et al., 2020). The original development and validation study (Buller et al., 2013) and the studies describing the updated version (Looney et al., 2018; Buller et al., 2020) contain a detailed description of how each model coefficient is derived and how all computation steps must be applied. For the application of the ECTemp™ algorithm on our data, ECTemp™ required a starting T_c_ and an associated variance that indicates the level of confidence of the starting T_c_ (Buller et al., 2013). We used a fixed starting core temperature of 37.0°C to estimate T_c−est_, as baseline core temperature values are typically unavailable for athletes exercising in a field setting. As we estimated the starting T_c_ we applied a starting variance of 0.02 (Buller et al., 2013). This way, the settings of the ECTemp™ algorithm reflected the regular use in daily practice.

### Statistical Analysis

Bland-Altman plots were generated by plotting the average of T_c−est_ and T_c_ values against the difference between the two methods. Bias was computed as the mean of the difference between T_c−est_ and T_c_, and a one-sample *t*-test was used to determine whether there was a systematic bias. To assess the agreement between T_c−est_ and T_c_, the limits of agreement (LoA) were derived from Bland-Altman plots (Bland and Altman, 1986), modified for multiple non-constant measurements per individual (Bland and Altman, 1999, 2007). The LoA were calculated for all data obtained during the exercise test, as well as for peak T_c_ data specifically. A bivariate correlation plot was constructed, and the Pearson correlation coefficient was calculated to further assess the agreement between T_c−est_ and T_c_. Furthermore, root mean square error (RMSE) weighted for participant and exercise test duration was computed (Buller et al., 2015). A one-way Analysis of Variance (ANOVA) was used to assess differences in the RMSE across subgroups of sex and sports discipline. Athletes were classified as endurance, mixed, power, or skill trained based on the relative isometric and isotonic components of their exercise training according to the European Society of Cardiology Guidelines (Pelliccia et al., 2021). The accuracy, sensitivity, and specificity of T_c−est_ to predict whether participants reached a certain core temperature value was examined using T_c−est_ classification thresholds between 37.0 and 39.75°C for all data and between 39.0 to 39.75°C for peak T_c_ data. We considered a sensitivity and specificity between 90 and 100% as excellent, 80–89% as good, 70–79% as fair, 60–69% as poor and <60% as failure to predict peak T_c_. Statistical analyses were performed using SPSS Statistics v25 (IBM Corp, Armonk, NY) and data were considered significant if *p* < 0.05. All parameters were visually inspected for normality and all data were presented as mean ± standard deviation (SD) unless indicated otherwise.

## Results

### Exercise Test Characteristics

**Figure 1:**
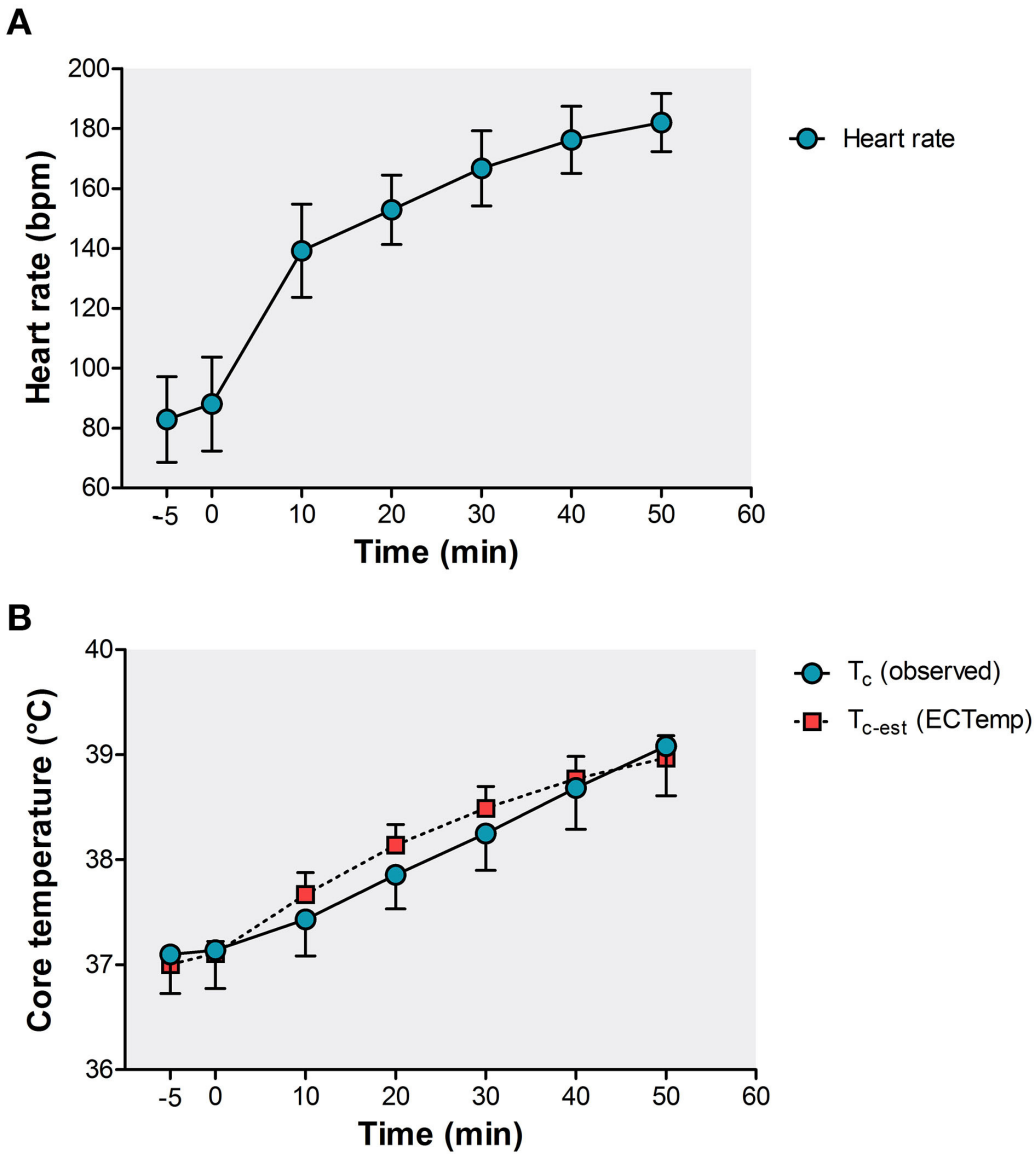
Exercise-induced responses in **(A)** heart rate and **(B)** observed T_c_ (temperature capsule, blue circles) and estimated T_c−est_ (ECTemp™ algorithm, red squares). Data is presented as mean ± SD.

The incremental exercise tests had a mean duration of 44 ± 10 min with a peak power output of 192 ± 53 W and a normalized peak power output of 2.6 ± 0.8 W/kg. Baseline HR was 82 ± 14 bpm and increased with 101 ± 15 bpm up to a peak HR value of 182 ± 12 bpm (Figure 1A). The range of HR was 45–207 bpm.

### Agreement Between T_c-est_ and T_c_

**Figure 2:**
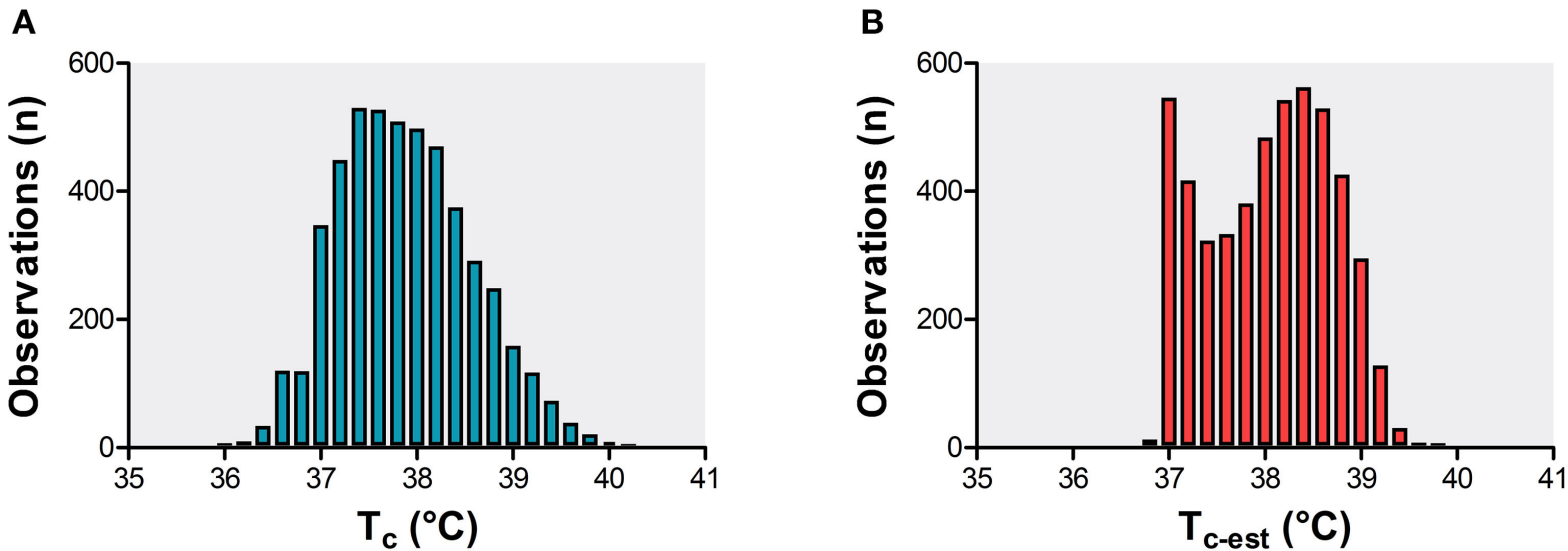
Frequency distribution plot of T_c_ observations **(A)** and computed T_c−est_ observations **(B)** of the analytical cohort (*n* = 101 elite athletes).

The dataset contained *n* = 5,025 T_c_ observations ranging from 35.8 to 40.4°C (Figure 2A). During the incremental exercise test, T_c_ increased from 37.1 ± 0.4°C at baseline to a peak T_c_ of 38.9 ± 0.6°C (Figure 1B). The computed T_c−est_ also consisted of *n* = 5,025 readouts and ranged from 36.7 to 39.9°C (Figure 2B). T_c−est_ increased to a peak value of 38.9 ± 0.3°C (Figure 1B).

**Figure 3:**
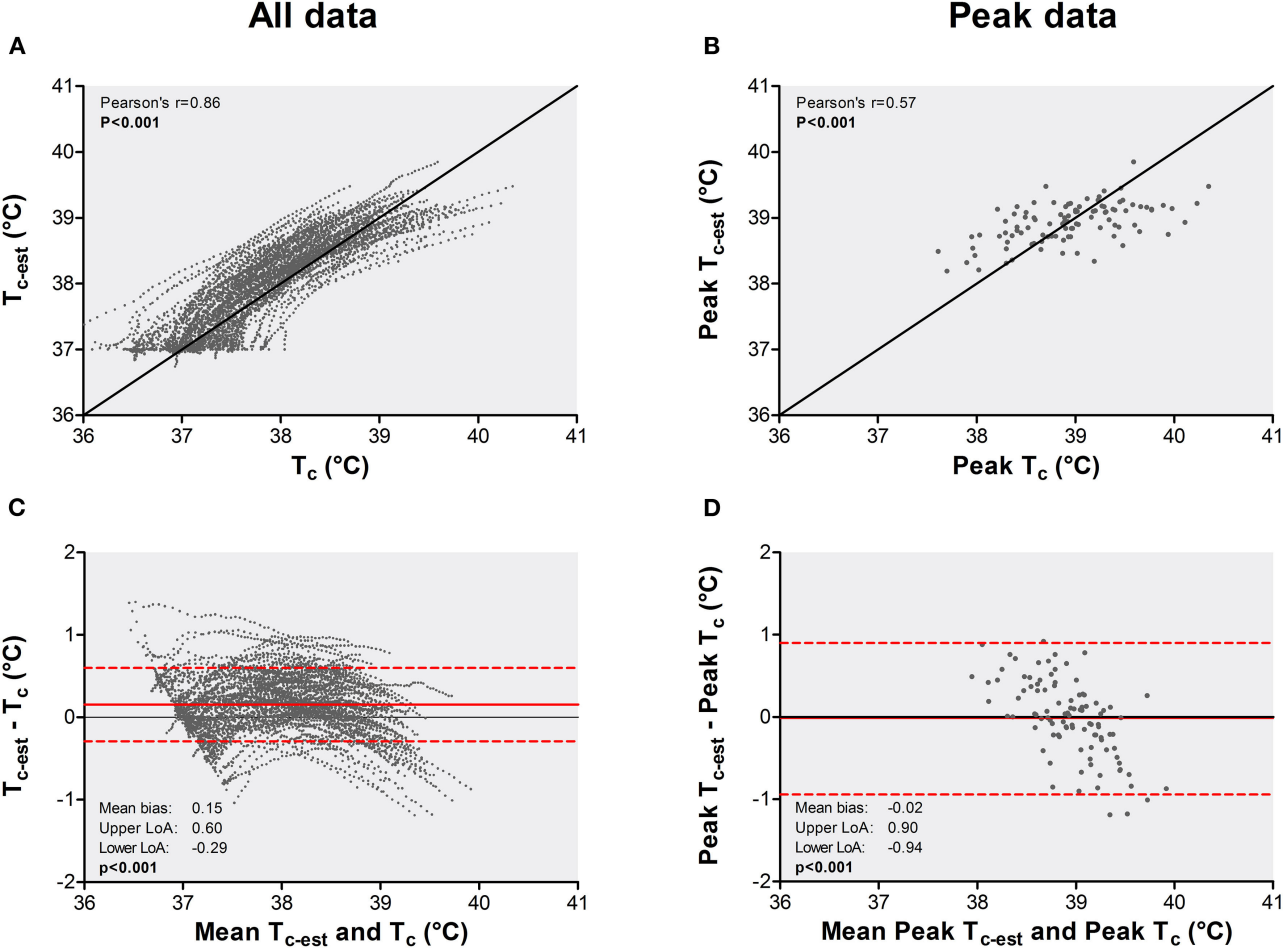
Bi-variate correlation and Bland-Altman plots for T_c−est_ vs. T_c_ across all data (panel **A,C**) and peak data (panel **B,D**). The bivariate correlations **(A,B)** are plotted with the line of identity as a solid black line. Data in Bland-Altman plots **(C,D)** is presented as mean difference (solid red line) and the upper and lower limits of agreement (dotted red lines).

A strong association was found between T_c−est_ and T_c_ (*r* = 0.86, *p* < 0.001, Figure 3A). A small yet significant systematic bias of 0.15 ± 0.36°C (*p* < 0.001) was found for T_c−est_, with LoA of ±0.45°C and a RMSE of 0.35 ± 0.18°C (Figure 3C). For peak T_c_ (range: 37.6–40.4°C), a moderate association between T_c_ and T_c−est_ was found (*r* = 0.57, *p* < 0.001, Figure 3B). A lower systematic bias (−0.02 ± 0.47°C (*p* < 0.001)) but greater LoA (±0.92°C) and RMSE (0.47°C) was found for T_c−est_ at peak T_c_ (Figure 3D).

**Figure 4:**
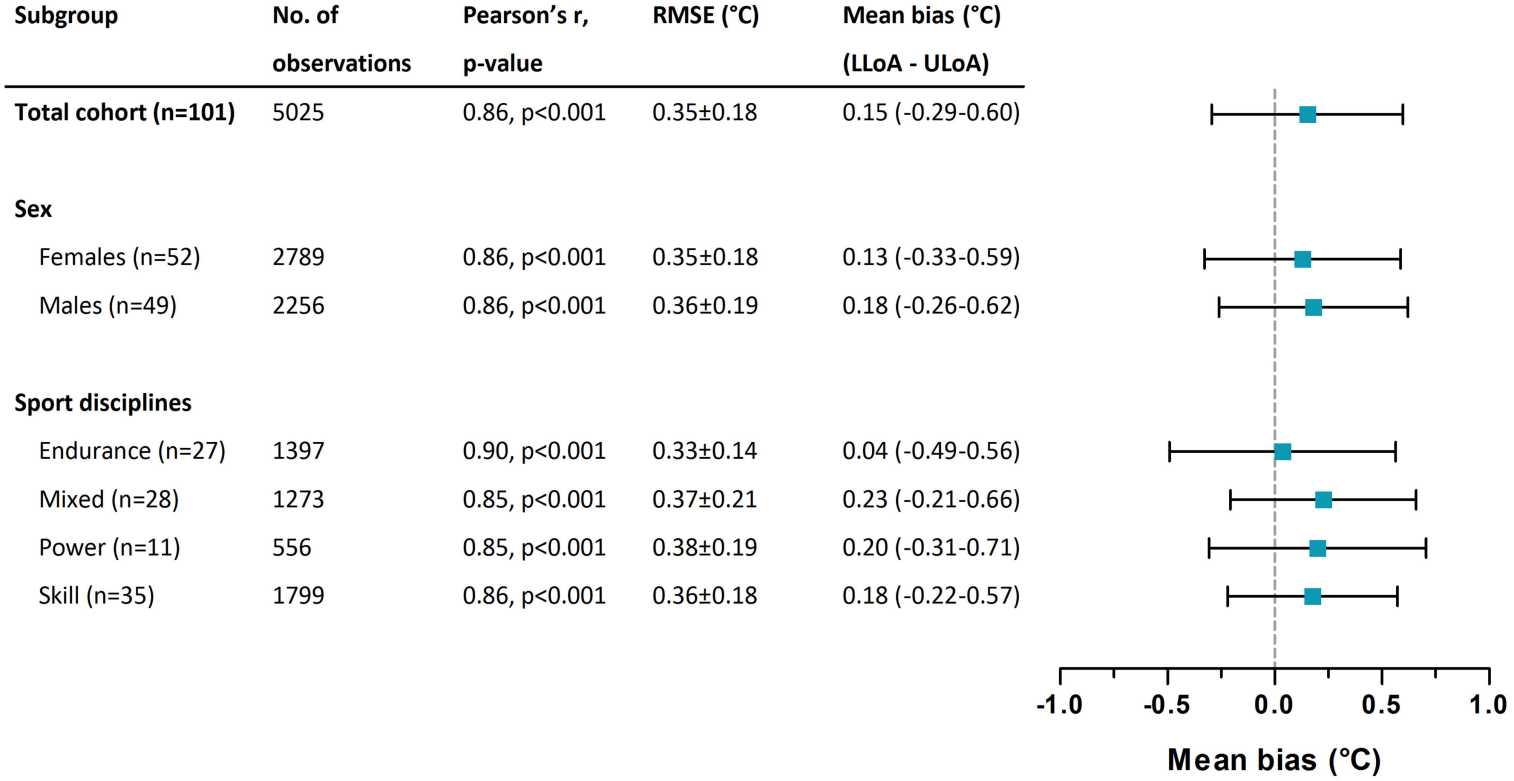
Subgroup-specific Pearson correlation coefficients between T_c−est_ and T_c_, root mean square errors (RMSE) weighted for participant and exercise test duration across T_c−est_ and T_c_, and the mean bias for T_c−est_. Mean bias is presented with the lower limit of agreement (LLoA) and the upper limit of agreement (ULoA).

The Pearson correlation coefficients between T_c−est_ and T_c_ ranged from 0.85 to 0.90 across subgroups based on sex and sport discipline (all *p*-values < 0.001, Figure 4). No differences were found for RMSEs across subgroups based on sex (*p* = 0.79) and sport discipline (*p* = 0.81, Figure 4). The mean bias for T_c−est_ varied between 0.04 and 0.23°C across subgroups, with the lowest mean bias for the endurance trained athlete group (Figure 4).

The accuracy of T_c−est_ varied between 84 and 99% (Table 2). The sensitivity was excellent (>90%) between 37.0 and 38.25°C but gradually declined with the lowest sensitivity (<16%) for T_c−est_ > 39.25°C (Table 2). The specificity of T_c−est_ demonstrated an opposite pattern and varied between 24 and 100%. The highest specificity (>90%) was observed for T_c−est_ values >38.75°C and the lowest specificity (<25%) for T_c−est_ values of 37.0°C (Table 2). The prevalence of false-negative observations was higher compared to true positive observations for all T_c−est_ thresholds >39.0°C. A comparable pattern for sensitivity and specificity was observed for peak core temperatures (Supplementary Table S1).

**Table 2:** Classification of T_c−est_ to predict core temperature value using T_c−est_ thresholds from 37.0 to 39.75°C.

**Total no. observations = 5,025**	**T**_**c−est**_ **threshold (°C)**											
	**>37.0**	**>37.25**	**>37.5**	**>37.75**	**>38.0**	**>38.25**	**>38.5**	**>38.75**	**>39.0**	**>39.25**	**>39.5**	**>39.75**
True positives (*n*)	4,466	3,767	3,231	2,654	2,035	1,411	878	456	163	22	3	0
False positives (*n*)	336	370	495	643	742	707	546	313	141	39	12	5
True negatives (*n*)	106	616	1,110	1,623	2,172	2,824	3,485	4,090	4,542	4,810	4,937	4,989
False negatives (*n*)	117	272	189	105	76	83	116	166	179	154	73	31
Accuracy (TP + TN/(P + N))	91%	87%	86%	85%	84%	84%	87%	90%	94%	96%	98%	99%
Sensitivity (TP/ (TP + FN))	97%	93%	94%	96%	96%	94%	88%	73%	48%	13%	4%	0%
Specificity (TN/(TN + FP))	24%	62%	69%	72%	75%	80%	86%	93%	97%	99%	100%	100%

*TP, true positives; TN, true negatives; P, positives; N, negatives; FN, false negatives; FP, false positives. Data is presented as n or %*.

## Discussion

We assessed the validity of the ECTemp™ algorithm to predict T_c_ of elite athletes performing exercise in simulated hot and humid environmental conditions. We observed a systematic bias of 0.15 ± 0.36°C, with LoA of ±0.45°C and a RMSE of 0.35 ± 0.18°C. These findings were not impacted by sex and/or sports discipline. We found a fair to excellent sensitivity (73–96%) and specificity (72–93%) for a T_c−est_ between 37.75 and 38.75°C, but the validity failed at T_c−est_ threshold >39.0°C and beyond due to a low to very-low sensitivity (50–0%). Findings from the current study show that the ECTemp™ algorithm can provide a valuable and representative indication of the thermal strain in the low- to mid-range of T_c_ values observed during exercise (37.75–38.75°C), but should not solely be used to identify athletes at risk for heat-related disorders due to the high false-negative rate and low sensitivity in the upper end of the T_c_ spectrum.

We observed a moderate to good validity for the ECTemp™ algorithm to predict T_c_ of elite athletes exercising in the heat, independent of sex and sport discipline, as illustrated by a strong correlation between T_c_ and T_c−est_ (*r* = 0.86, *p* < 0.001), low systematic bias (0.15 ± 0.36°C), reasonable LoA ±0.45°C and RMSE 0.35 ± 0.18°C, and good to excellent diagnostic accuracy (84–99%). These outcomes align with previously reported correlations (*r* = 0.84 to *r* = 0.91), biases (−0.28 to 0.34°C), LoAs (0.48–0.78°C), and RMSEs (0.21–0.49°C) observed in military personnel (Buller et al., 2013, 2015, 2020; Looney et al., 2018), and indicate that the ECTemp™ algorithm may be used beyond the application it was initially developed for. However, although these validity indices were based on a large number of observations in the full dataset, it is important to note that these outcomes may not apply to the full range of T_c_ measurements. Indeed, a less favorable correlation (*r* = 0.57, *p* < 0.001), LoA (±0.92°C), RMSE (0.47°C) and diagnostic accuracy (72–91%) were found for peak T_c_ data. These findings indicate that the validity of the ECTemp™ algorithm is dependent on the magnitude of exercise-induced increases in T_c_.

Utilizing the ECTemp™ algorithm as a diagnostic test resulted in fair to excellent sensitivity (73–96%) and specificity (72–93%) for T_c−est_ values between 37.75 and 38.75°C. However, the diagnostic accuracy was substantially reduced at T_c−est_ threshold >39.0°C and beyond. The higher prevalence of false-negative compared to true positive observations led to a low to very-low sensitivity (50–0%). These observations align with a previous ECTemp™ study that reported the lowest sensitivity (<40%) for the highest T_c_ values (>40.0°C) (Buller et al., 2020). The poor validity to estimate high T_c_ values was also reported by another ECTemp™ study during treadmill exercise whilst wearing personal protective equipment (Hunt et al., 2019). Differences between T_c_ and T_c−est_ were found to be greater for the highest T_c_ values (>38.5°C) (Hunt et al., 2019). A potential explanation for the poor performance of the algorithm at the upper end of the T_c_ spectrum may relate to the assumption of a fixed relationship between the cardiovascular and thermoregulatory system captured by two single parameters (i.e., HR and T_c_) (Buller et al., 2020). Therefore, if the HR – T_c_ relationship changes, for example due to the redistribution of blood flow and maintenance of blood pressure following dehydration, ECTemp™ will likely underestimate or overestimate T_c_ (Buller et al., 2020). Given the large underestimations in the upper end of the T_c_ spectrum, it thus seems that the use of a single input parameter (i.e., HR) contains insufficient information to reflect the cumulative thermoregulatory and cardiovascular effects of prolonged exercise in the heat (Periard et al., 2021). A potential solution to overcome this problem may be to calibrate the algorithm against T_c_ measurements of the individual athlete during exercise training sessions. We have previously shown that within-participant variations in exercise-induced T_c_ responses are small during a mass-participation road running race (Veltmeijer et al., 2015). Therefore, we hypothesize that implementing individual information of previously obtained T_c_ responses into the ECTemp™ algorithm may improve its performance.

Taken together, the ECTemp™ algorithm can provide an accurate indication of thermal strain for T_c_ values between 37.75 and 38.75°C, and therefore seems sufficiently valid for heat acclimatization purposes in which a target T_c_ (e.g., 38.5°C) is attained and maintained for a given time (Periard et al., 2015; Daanen et al., 2018). However, the high false-negative rate and low sensitivity in the upper end of the T_c_ spectrum emphasize that the algorithm, in its current form, should not solely be used to identify athletes at risk for heat-related disorders (Epstein and Roberts, 2011). Therefore, the validity of the ECTemp™ algorithm for athletes depends on the goal of the end-user.

### Strengths, Limitations and Future Perspectives

We assessed the validity of the ECTemp™ algorithm in a unique and large group of 101 elite athletes exercising in the heat, using a well-controlled exercise protocol. Another strength of our study is the large number of T_c_ readouts (*n* = 5,025) allowing validation of the algorithm across a large T_c_ spectrum (35.8–40.4°C). Outcomes of our study can be of interest to athletes and coaches as the ECTemp™ algorithm may be used in regular training sessions with an expected T_c_ in the low to mid-range, or while evaluating heat preparedness strategies such as cooling interventions and monitoring physiological adaptations during heat acclimation. A limitation of our study is the use of an ingestible telemetric temperature capsule system as the reference measurement and surrogate marker for T_c_. Although the myTemp system has been demonstrated to be valid (Bongers et al., 2018a,b), an intestinal sensor may respond less rapidly compared to the esophageal temperature at the start of exercise or to a change in exercise intensity (Byrne and Lim, 2007), resulting in an underestimation from the actual T_c_ and possibly even larger underestimation of T_c−est_ at any given time point. We have only examined the validity of ECTemp™ in a specific setting, namely during a laboratory-based incremental cycling protocol in hot and humid conditions, resulting in a gradual and progressive increase in T_c_. As the T_c_ response may be very different in real-life field conditions (in terms of magnitude and the course over time), future studies are warranted to examine whether the performance of the algorithm is reproducible under field conditions. Such follow-up studies can also determine whether the ECTemp™ validity is reproducible across different exercise modalities (i.e., continuous vs. intermittent exercise) and environmental conditions (i.e., cool vs. moderate vs. hot). Another important consideration for future work is that most of our T_c_ observations are in the mid-range of the T_c_ spectrum. Especially higher T_c_ values are underrepresented. Although our findings are robust, additional research into the performance of the algorithm in the upper end of the T_c_ spectrum is warranted.

## Conclusion

The findings from our study show that the ECTemp™ algorithm should in its current form, not solely be used to identify (elite) athletes at risk for heat-related disorders due to low sensitivity and high false-negative rate in the upper end of the T_c_ spectrum. However, the ECTemp™ algorithm can provide a valuable and representative indication of thermal strain in the low- to mid-range of T_c_ values (37.75–38.75°C) observed in elite athletes during relatively short (44 min on average) incremental exercise in the heat. It may, therefore, be a useful non-invasive and non-obtrusive tool to inform athletes and coaches about the estimated core temperature during controlled hyperthermia heat acclimation protocols.

## Data Availability Statement

The data that support the findings of this study are available from the corresponding author upon reasonable request.

## Ethics Statement

The study was in accordance with the Declaration of Helsinki and was approved by the Medical Ethical Committee of the Radboud university medical center (#2018-4640). All participants gave their written informed consent prior to the testing procedures.

## Author Contributions

JK and TE were involved in the study design and protocol conception. JK, CB, and TE were involved in the data collection. JK, BV, and ED processed the physiological data. JK analyzed the data and was responsible for the initial writing and drafting of the article. All authors critically revised the manuscript and approved the final version of the manuscript.

## Funding

This work was funded by a ZonMW Grant (#546001003) as part of the Thermo Tokyo project.

## Conflict of Interest

The authors declare that the research was conducted in the absence of any commercial or financial relationships that could be construed as a potential conflict of interest.

## Publisher's Note

All claims expressed in this article are solely those of the authors and do not necessarily represent those of their affiliated organizations, or those of the publisher, the editors and the reviewers. Any product that may be evaluated in this article, or claim that may be made by its manufacturer, is not guaranteed or endorsed by the publisher.
