# Supplementary material for: A Heart Rate Based Algorithm to Estimate Core Temperature Responses in Elite Athletes Exercising in the Heat
Source: Front Sports Act Living. 2022 Jun 22;4:882254. doi: 10.3389/fspor.2022.882254 (PMC9256956; doi:10.3389/fspor.2022.882254)
Supplement: Supplementary file 1 [file Table_1.DOCX]

**Supplementary Table 1**. Classification of T_c-est_ to predict peak core temperature values using T_c-est_ thresholds from 39.0 to 39.75.

| Total no. observations = 100 | **T_c-est_ threshold (°C)** | | | |
| --- | --- | --- | --- | --- |
|  | **>39.0** | **>39.25** | **>39.5** | **>39.75** |
| **True positives (n)** | 30 | 5 | 1 | 0 |
| **False positives (n)** | 14 | 4 | 0 | 1 |
| **True negatives (n)** | 42 | 65 | 85 | 91 |
| **False negatives (n)** | 14 | 26 | 14 | 8 |
| **Accuracy (TP + TN/(P + N))** | 72% | 70% | 86% | 91% |
| **Sensitivity (TP/ (TP + FN))** | 68% | 16% | 7% | 0% |
| **Specificity (TN/ (TN + FP))** | 75% | 94% | 100% | 99% |
| TP, true positives; TN, true negatives; P, positives; N, negatives; FN, false negatives; FP, false positives. Data is presented as n or %. | | | | |
